# Organelle-Specific Mechanisms in Crosstalk between Apoptosis and Ferroptosis

**DOI:** 10.1155/2023/3400147

**Published:** 2023-01-05

**Authors:** Peiyao Wu, Xiaoyue Zhang, Dingyu Duan, Lei Zhao

**Affiliations:** ^1^Department of Periodontics, West China Hospital of Stomatology, Sichuan University, Chengdu, China; ^2^State Key Laboratory of Oral Diseases and National Clinical Research Center for Oral Diseases, West China Hospital of Stomatology, Sichuan University, Chengdu, China

## Abstract

Apoptosis has been extensively studied, whereas ferroptosis is a newly discovered form of regulated cell death that involves iron-dependent accumulations of lipid hydroperoxides. While these two cell death mechanisms were initially believed to be mutually exclusive, recent studies have revealed cellular contexts requiring a balanced interaction between them. Numerous subcellular sites and signaling molecules within these sites are involved in both processes, either as modules or switches that allow cells to choose on how to proceed. The close relationships between apoptosis and ferroptosis, as well as the possibility of switching from one to the other, are described in this review. To understand the crosstalk between apoptosis and ferroptosis, various organelle-specific mechanisms must be analyzed and compared. The ability to switch apoptosis to ferroptosis by targeting cellular organelles has a great potential in cancer therapy.

## 1. Introduction

Cell death is closely related to organism growth, development, aging, and diseases. Based on controllability, cell death can be classified into two types: programmed cell death (PCD) and nonprogrammed cell death. To date, PCD includes apoptosis, necroptosis, pyroptosis, and ferroptosis, among others [1].

Apoptosis is the most common and best-studied PCD type. External and internal stimuli, in combination with extrinsic and intrinsic apoptosis pathways, can initiate apoptosis. Both these pathways ultimately activate cysteine aspartate proteases (caspases) [2]. Caspases are cysteine-dependent aspartate-specific proteases that cleave hundreds of different proteins during apoptosis, formation of an atypical morphology in apoptotic cells, such as cell rounding, chromatin condensation (which results in nuclear pyknosis), nuclear fragmentation, shedding of apoptotic bodies, cytoplasm-containing vacuoles, and intact organelles [3]. Intrinsic apoptosis is triggered by mitochondrial outer membrane permeabilization (MOMP), which allows soluble proteins such as cytochrome c (Cyt c) to permeate into the cytosol from the mitochondrial intermembrane gap. When Cyt c oligomerizes apoptotic protease activating factor-1 (APAF-1), Cyt c triggers the formation of a caspase-activating complex, the apoptosome. Caspase-3 and -7 are then activated, cleaving multiple substrates to trigger apoptosis by binding to and activating the caspase-9 initiator [4]. This process is primarily mediated by the B-cell lymphoma-2 (Bcl-2) protein family. Extrinsic stimuli can also induce apoptosis through cell surface death receptors including TNF (tumor necrosis factor), Fas (CD95/APO1), and TNF-related apoptosis-inducing ligand (TRAIL) [5]. Extrinsic apoptosis can induce intrinsic apoptosis through caspase-8-mediated activation of the Bcl-2 family member BID [6].

Recent discoveries have found that ferroptosis is a new form of PCD. While screening for antitumor drugs, Dolma et al. [7] discovered that erastin caused cancer cell death. Interestingly, in contrast to what was known then, erastin-induced cell death was novel, with no observed formation of apoptotic vesicles, DNA breaks, or activation of the caspase family, and was not reversed by caspase inhibitors. After intensive research, Dixon et al. [8] further proposed a concept of iron-dependent PCD in 2012 and formally named it ferroptosis. Ferroptosis is accompanied by intracellular iron ion disorder, lipid peroxide accumulation, and abnormal metabolism of glutathione (GSH), which result in membrane breakage and cell death through lipid peroxidation on the inner side of cell membrane phospholipids [9]. Iron uptake, secretion, utilization, and storage are all interrelated to intracellular iron homeostasis [10]. Fe^3+^ enters and exits the cell via transferrin receptor 1 (TFR1) and TFR2, and because of the action of enzymes, it becomes an unstable Fe^2+^ and is stored in the cytoplasmic iron pool. Once the TFR function is abnormal, Fe^3+^ uptake increases or excretion decreases, resulting in intracellular Fe^3+^ accumulation. Excess Fe^2+^ generates hydroxyl radicals (•OH)—a reactive oxygen species (ROS)—through the Fenton reaction, thereby inducing ferroptosis [11]. Lipid peroxidation involves the oxidative breakdown of polyunsaturated fatty acids (PUFA) and lipids in the cell, resulting in the production of unstable lipid moieties that can damage mitochondrial membranes and cell membranes and thus greatly affect cell function [12]. GSH is the main substrate of glutathione peroxidase-4 (GPX4) with a protective effect on cells. GPX4 binds to lipid peroxides and reduces ROS, thereby attenuating the ROS-induced damage to cells. System Xc-, an intracellular antioxidant system, plays a role in GSH synthesis. When intracellular cysteine is depleted or system Xc- is inhibited, GSH production decreases and intracellular levels of ROS and lipid peroxides increase, thus promoting ferroptosis [13].

Distinct modes of PCD have long been studied in isolation, as widely used models suggested that they represent mutually exclusive cellular states. Numerous studies have recently shown a correlation between ferroptosis and other cell death processes such as apoptosis and necroptosis. For example, Müller et al. [14] demonstrated that ferroptosis and necroptosis have a synergistic effect on tissue damage during acute organ failure *in vivo*. Necroptosis appears to function in the enhanced susceptibility of cystic fibrosis airway epithelial cells to ferroptosis [15]. Ferroptosis and apoptosis differ in terms of cell morphological characteristics, biochemical features, and other features, as outlined in Table 1. However, a growing body of evidence indicates that ferroptosis and apoptosis can interact cooperatively or be used by cells in a complementary manner to facilitate cellular destruction, depending on the cellular environment and death triggers. Similar pathways, subcellular sites, and organelles are involved in their regulation. This review briefly describes the role that organelles play in ferroptosis–apoptosis interactions.

## 2. Crosstalk between Ferroptosis and Apoptosis

Cell death has a crucial role in diseases. Numerous studies have demonstrated the role played by apoptosis in disease development. For example, studies have shown that in neurodegenerative diseases such as Alzheimer's and Parkinson's diseases, neuronal cell death often occurs through apoptosis [16]. Renehan et al. [17] found that apoptosis of intestinal epithelial cell damages the epithelial barrier and is involved in ulcerative colitis development. Apoptosis of vascular endothelial cells can trigger endothelial cell dysfunction, thereby initiating atherosclerosis [18]. Recent studies have found that ferroptosis also has a crucial function in diseases. Ferroptosis is also an important cell death pathway in neuronal cells, and the ferroptosis inhibitor ferrostatin-1 (Fer-1) inhibits neuronal cell death *in vitro* and *in vivo* and reduces disease severity [19, 20]. Xu et al. [21] found that intestinal epithelial cells in ulcerative colitis undergo ferroptosis. Bai et al. [22] showed that endothelial cells undergo ferroptosis in atherosclerosis, resulting in endothelial dysfunction; this process can be reversed by Fer-1. Although these studies have shown the importance of apoptosis and ferroptosis in diseases, the results equally demonstrated that inhibition of apoptosis or ferroptosis does not completely inhibit cell death, suggesting that these two modes of death cooperate with each other in diseases, in addition to the possible involvement of other cell death modes. Moreover, further in-depth investigation is required to determine whether a temporal sequence and possible regulatory factors exist for the same type of cells that may undergo apoptosis as well as ferroptosis during the development of the same disease.

Several studies have suggested that apoptosis is converted to ferroptosis under certain conditions and that ferroptosis promotes cellular susceptibility to apoptosis. The tumor suppressor p53 is activated by apoptosis-stimulating proteins, thereby triggering apoptosis as a response to DNA damage. p53-induced apoptosis is inhibited by the inhibitor of the apoptosis-stimulating protein of p53 (iASPP), thereby promoting tumor growth. iASPP-overexpressing human cancer cells can exhibit chemoresistance [23]. Li et al. [24] found that iASPP overexpression inhibited ferroptosis, while ferroptosis was promoted with the knockdown of this protein *in vivo*, suggesting that p53 may be a key molecule in the ferroptosis–apoptosis crosstalk. Furthermore, the apoptosis-inducing factor (AIF) is initially involved in caspase-independent apoptosis [25]. After nuclear translocation, AIF promotes ferroptosis in mouse hippocampal HT22 or MEF cells [26, 27]. PUFA are vital components of cell membrane phospholipids. PUFA exogenously delivered to cells led to marked accumulation of PUFA phospholipids and increased sensitivity to oxidative stress, thus decreasing viability [28]. However, despite an increase in apoptotic corpses in dihomogamma-linolenic acid- (DGLA-) induced germ cell death, DGLA can still induce cell death and sterility in apoptosis-deficient mutants [29].

According to Perez et al. [30], Fer-1-sensitive mechanisms, along with apoptosis, can trigger germ cell death. DGLA-exposed apoptosis-deficient mutants were completely sterile when Fer-1 was administered, indicating that both ferroptosis and apoptosis are activated as a response to DGLA. Furthermore, their findings suggested that ferroptosis and apoptosis are mutually exclusive, as resistance to one pathway did not protect the cells from death via the other pathway. Furthermore, erastin is frequently believed to be a ferroptosis activator. Several studies, however, have shown that erastin can also cause apoptosis. Huang et al. [31] demonstrated that erastin-induced p53 promotes A549 lung cancer cell apoptosis and inhibits cell proliferation. According to Huo et al. [32], erastin is potently cytotoxic to various colorectal cancer cell lines in part by inducing oxidative stress and caspase-9-dependent cell death. The primary symptom of ferroptosis progression is intracellular GSH depletion. Curcumin causes GSH depletion, thereby triggering caspase-dependent and caspase-independent apoptosis in mouse fibroblast cells and human leukemic cells [33, 34]. The mechanism through which ferroptosis and apoptosis interact is unknown. Both the apoptosis inhibitor (Z-VAD) and ferroptosis inhibitors (Fer-1 and liproxstatin-1) can prevent quercetin-induced cell death [35]. Fer-1 and Z-VAD can both inhibit caspase-9 and PARP cleavage. However, their combination showed no significant prevention of further cleavage, indicating that ferroptosis occurs prior to apoptosis. All these findings highlight the link between apoptosis and ferroptosis and raise concerns about the theoretical basis of ferroptosis and the ferroptosis–apoptosis.

## 3. Role of Different Organelles in Apoptosis and Ferroptosis

During cell death, a series of intracellular physiological and biochemical reactions occur. Some of these reactions occur in the cytoplasm, while some occur in the organelles composed of the endosomal system. However, they work together in a certain spatial and temporal sequence to complete the cell death process. Structural or functional alterations occur in organelles such as mitochondria, endoplasmic reticulum (ER), lysosomes, Golgi apparatus (GA), and peroxisomes during apoptosis and ferroptosis (Table 2). However, the role of these organelles and their interactions in the apoptosis–ferroptosis crosstalk remain unclear.

### 3.1. Mitochondria

#### 3.1.1. Mitochondria and Apoptosis

Since the discovery of apoptosis, many studies have explored the role of mitochondria in this PCD type. Several recent studies have shown that mitochondria are critical for apoptosis.

Newmeyer et al. [36] first proposed the link between mitochondria and apoptosis by using the cell-free system of Xenopus egg extracts. They showed that mitochondria with a dense organelle fraction could result in chromatin condensation and nuclear fragmentation, thus indicating a role for mitochondria in apoptosis. Follow-up studies revealed that several factors involved in the mitochondrial apoptotic signaling pathway localize in the mitochondria. Under various stimuli—such as hypoxia, excess ROS, DNA damage, and lack of cytotropic factors—cells exhibit disruption of the mitochondrial membrane potential homeostasis, increased permeability, and release of mitochondrial proapoptotic factors into the cytoplasm, which thereby activate the mitochondrial apoptosis pathway and cause cell death. Proapoptotic factors include Cyt c, AIF, APAF-1, and adenylate kinase 2 (AK2) [37, 38]. Cyt c is a crucial mitochondrial component as it is a key player in the life-sustaining process of ATP synthesis. When apoptosis is triggered, Cyt c is released into the cytosol [39]. AIF can also function in the mitochondria as a FAD-dependent NADH oxidase. When released from the mitochondria, it migrates to the nucleus in response to apoptotic stimuli, where it induces chromatin condensation and DNA breakage. This is an apoptotic pathway that functions without caspases [40]. Lee et al. [41] suggested that AK2 is a component of the AK2–FADD–caspase-10 complex that may contribute to tumorigenesis through a novel intrinsic apoptotic pathway.

The release of proapoptotic factors is believed to occur following the opening of the permeability transition pore (PTP) channel, which includes the voltage-dependent anion channel (VDAC), adenine nucleotide translocator, cyclophilin D, and other molecules [42]. When the PTP opens, mitochondrial membrane permeability increases, mitochondria swell, and the outer mitochondrial membrane ruptures; this process is mediated by the Bcl-2 protein family. Most Bcl-2 family proteins are localized on the mitochondrial membrane, similar to antiapoptotic proteins Bcl-2, Bcl-xL, and Mcl-1 and proapoptotic proteins Bad, BID, and Bik [43]. A study showed that Bcl-2 inhibits PTP basal activity, affecting the mitochondrial membrane permeability of normal cells [44]. Bad, a protein that promotes apoptosis, increases the sensitivity of mitochondrial PTP to Ca^2+^ through a Bcl-xL-sensitive and VDAC-mediated process [45]. In addition, mitochondrial involvement in apoptosis is also related to its impaired energy metabolism. Mitochondrial respiratory chain complexes are crucial for driving various biochemical processes in eukaryotic cells by producing energy through oxidative phosphorylation [46]. Rotenone, a mitochondrial respiratory chain inhibitor, and oligomycin, a mitochondrial ATP-synthase inhibitor, could induce apoptosis in cultured human lymphoblastoid and other mammalian cells, which is not inhibited by Bcl-2 [47]. Rotenone can increase mitochondrial ROS (mitoROS) production by inhibiting mitochondrial respiratory chain complex I, thereby inducing apoptosis [48]. Mitochondrial DNA (mtDNA) encodes mitochondrial respiratory chain complexes. Generally, a mtDNA mutation leads to a loss of functionality of the oxidative phosphorylation system and thus to ATP depletion and ROS overproduction, which induce apoptosis [49]. Accumulation of selective mtDNA mutations leads to beta-cell apoptosis and diabetes development [50].

#### 3.1.2. Mitochondria and Ferroptosis

The role of mitochondria in ferroptosis is currently under debate. Ferroptotic cells exhibit swollen mitochondria with reduced cristae, dissipation of mitochondrial membrane potential, and increased mitochondrial membrane permeability [8], thereby indicating mitochondrial dysfunction. Gao et al. demonstrated that although mitochondria are crucial for erastin-induced or cysteine-deprivation-induced ferroptosis, they are not required for RSL3-induced ferroptosis [51]. The role of mitochondria in ferroptosis is now believed to be related to the cell type, the regulatory signaling network induced by different drugs.

Energy metabolism also plays a major role in ferroptosis. Glycolysis, tricarboxylic acid (TCA) cycle, and mitochondrial electron transport chain (ETC) respiratory pathways are required for energy provision in cells and various other physiological functions [52]. Switching of metabolism from mitochondrial respiration to aerobic glycolysis makes maintaining of cellular energy under pathophysiological conditions easier. However, in erastin-induced ferroptosis, an increase in ATP and oxidative phosphorylation synthesis reduces glycolysis flux [53]. Wang et al. [54] found that RSL3, a type of ferroptosis activator that can inactivate GPX4, causes glioma cell death by disrupting glycolysis. Switching to oxidative phosphorylation makes cells with disrupted glycolysis susceptible to ferroptosis [55], whereas Krabbendam et al. [56] discovered that shifting mitochondrial energy metabolism to glycolysis offered beneficial antiferroptosis effects to neuronal cells. In addition, inhibition of the classical metabolic activity, including mitochondrial TCA cycle and ETC activity, results in the inhibition of cysteine deprivation-induced ferroptosis [57]. Furthermore, aconitase, citrate synthase, fumarate hydratase (FH), and other TCA cycle enzymes are associated with ferroptosis through mitochondrial respiration [51, 58]. FH deficiency protects against erastin-induced ferroptosis [51].

Along with being an energy factory, in most mammalian cells, mitochondria mainly produce ROS. MitoROS may be one mechanism through which mitochondria are involved in ferroptosis promotion. Increased mitochondrial production is a major contributor to a glutamate-induced cell death model [59]. By contrast, DeHart et al. [60] proposed that erastin and its analogs induce ferroptosis, resulting in increased mitochondrial and cytoplasmic ROS production. They also proposed that both the mitochondria-targeted antioxidant MitoQ and the ROS scavenger N-acetylcysteine prevented mitochondrial and cytoplasmic ROS production, thereby rescuing mitochondrial function and cell viability. Another study synthesized XJB-5-131, a mitoROS-targeting scavenger, and found it to be more effective in inhibiting cellular ferroptosis than a broad range of ROS scavengers [61]. Interestingly, RSL3 exposure clearly resulted in a significant loss of GPX4 expression and increased lipid peroxidation, despite MitoQ protecting the mitochondrial function, integrity, and cell viability. This implies that mitochondrial damage may be the last stage of ferroptosis [62].

Mitochondria are also the primary organelle for intracellular iron regulation. Erastin-induced ferroptosis is considered to increase mitochondrial Fe^2+^ [63]. Because increased levels of mitochondrial labile iron infer increased ROS accumulation in ferroptosis, an increased level of mitochondrial iron storage may help prevent ferroptosis. Mitochondrial ferritin (FtMt) influences iron metabolism by modifying the redistribution of iron from the mitochondria to the cytosol and inhibiting oxidative stress-induced cell damage as an iron-storage protein, particularly in tissues with high oxygen consumption [64]. FtMt significantly reduced the cellular labile iron pool, ROS, and subsequent erastin-induced ferroptosis [65].

#### 3.1.3. The Role of Mitochondria in Apoptosis–Ferroptosis Crosstalk

**Figure 1:**
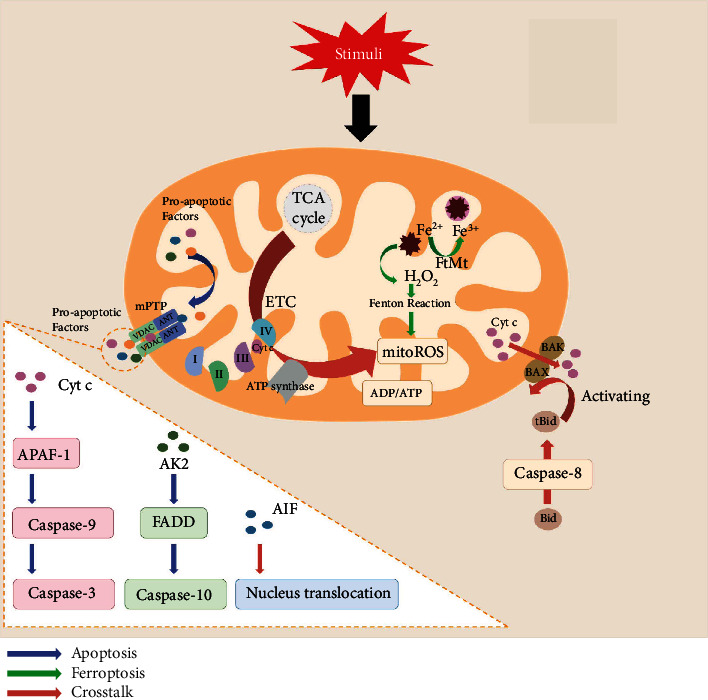
Role of mitochondria in apoptosis and ferroptosis.

The mitochondrial pathway is crucial for both apoptosis and ferroptosis (Figure 1). Whether it is apoptosis or ferroptosis, mitochondrial membrane permeability plays a decisive role in determining the survival or death of cells, indicating the significance of mitochondria in the two PCD types. For example, mitochondrial depletion reduced abivertinib-induced cytotoxicity, including ferroptosis and apoptosis [66]. Furthermore, Bim- and Bax-mediated mitochondrial pathways not only influence apoptosis but also act upstream of SLC7A11 and GPX4 to mediate AC-induced ferroptosis. At this level, the mitochondria establish a battlefield on which signals combat to seal the cell fate. Some mitochondria-derived apoptosis regulators also have a regulatory role in ferroptosis. In addition to that the aforementioned transport of AIF from the mitochondria to the nucleus induces ferroptosis, according to a recent study, BID links ferroptosis to mitochondrial apoptosis. This protein connects surface death receptors to the mitochondrial core extrinsic apoptotic pathway. Caspase-8 activates it, resulting to its entry into the mitochondria, where BID activates both BAX and BAK, ensuring the release of Cyt c. In neuronal cells, erastin-induced ferroptosis is characterized by BID transactivation to mitochondria, loss of mitochondrial membrane potential, increased mitochondrial fragmentation, and decreased ATP levels. A BID inhibitor, BI-6c9, can prevent erastin-induced ferroptosis [67]. BI-6c9 and the CRISPR/Cas9 BID knockout are protected against RSL3 toxicity, restoring mitochondrial integrity and function [62]. BID, according to Wang et al. [35], is a critical mediator for the apoptosis–ferroptosis crosstalk. Despite previous findings that apoptosis inhibitors are ineffective against ferroptosis, further investigation is required to understand the specific mechanistic role of BID in ferroptosis.

Impairment of mitochondrial energy metabolism has a critical role in both apoptosis and ferroptosis mechanisms. Both apoptosis and ferroptosis induce mitochondrial dysfunction in ETC, which results in reduced ATP levels and excess ROS production [48, 68]. The ADP/ATP ratio can be used to determine cell viability, necrosis, and apoptosis [69]. A decrease in the cellular energy status activates AMPK, signaled by high ADP/ATP ratios. Cancer cells with a high basal AMPK activation level can resist ferroptosis, whereas inactivating AMPK makes these cells prone to ferroptosis [70]. Thus, the ADP/ATP ratio may act as a signal determining whether cells undergo apoptosis or ferroptosis.

### 3.2. ER

#### 3.2.1. ER and Apoptosis

The ER is essential to protein folding and trafficking into the secretory pathway. Environmental insults or increased protein synthesis frequently results in protein misfolding in the organelle. In the absence of ER protein folding, a cellular stress response known as ER stress is induced. Cells die when protein misfolding is not resolved. During prolonged ER stress, signaling via protein kinase R-like ER kinase (PERK), activating transcription factor 6 (ATF6), and inositol-requiring enzyme-1 (IRE1) triggers proapoptotic signals. They start the activation of downstream molecules such as the C/EBP-homologous protein (CHOP), which leads to cell death [71].

As unfolded proteins accumulate, PERK and IRE1 are dissolved and activated via autophosphorylation [72]. PERK and IRE1 pathways may converge on CHOP, potentially amplifying each other's effects [73]. ER stress-induced apoptosis is partially protected by CHOP deficiency in CHOP^−/−^ mouse embryonic fibroblasts, implying that CHOP has a role in this apoptosis process [74]. At the transcriptional level, CHOP can induce apoptosis by downregulating the antiapoptotic protein Bcl-2 and upregulating proapoptotic proteins such as the Bcl-2-interacting mediator of cell death (BIM) and p53-upregulated modulator of apoptosis (PUMA) [75]. These proapoptotic proteins can activate the transcription of several genes, resulting in caspase activation and ultimately cell death [76]. Proteolytic enzymes in the GA also activate ATF6. Activated ATF6 molecules increase caspase-3 transcriptional activity by upregulating downstream CHOP molecules [77]. During late luteal regression, ATF6 performs crucial apoptotic functions in rats via ATF6/CHOP and caspase-12 pathways [78]. Melatonin also protects against neuronal apoptosis in an intracerebral hemorrhage rat model by suppressing the ATF6/CHOP pathway [79].

The ER is not only involved in protein synthesis and maturation but also serves as a calcium (Ca^2+^) reservoir to maintain intracellular Ca^2+^ homeostasis. Numerous experiments have shown that in the early apoptosis stages, many cells exhibit a rapid and sustained increase in cytosolic Ca^2+^ concentration, which is because of Ca^2+^ release from the ER [80–82]. On the one hand, increased Ca^2+^ concentration activates calpain, which in turn activates procaspase-12 [83]. Active caspase-12 released from the ER membrane moves into the cytosol and activates procaspase-9, thereby further activating executive caspase-3. Additionally, this pathway operates independent of APAF-1 and does not require Cyt c release from mitochondria [84]. The released Ca^2+^ that enters the cytosol, on the other hand, is rapidly captured and accumulated within mitochondria, thereby depolarizing the inner mitochondrial membrane and inducing mitochondria-dependent apoptosis [85].

#### 3.2.2. ER and Ferroptosis

Fer-1, a ferroptosis inhibitor, may exhibit its antiferroptotic effect by accumulating in the ER rather than in the lysosomes and mitochondria [86]. Quantitative measurements suggest that the ER viscosity increases during erastin-induced ferroptosis [87]. Findings of this study indicate that the ER is involved in ferroptosis.

Recent evidence indicates that the ER stress response is vital in ferroptosis. Dixon et al. [88] discovered that erastin treatment activated ER stress and induced ferroptosis in HT-1080 cells. RNA sequencing revealed ER stress-related changes such as phosphorylation of translation initiation factor 2 (eIF2) and upregulation of ATF4 protein levels compared to unphosphorylated forms and total protein. Similarly, dihydroartemisinin induced ferroptosis in glioma cells through the PERK/ATF4/HSPA5 pathway [89]. According to these findings, ferroptosis activators induce ferroptosis in many cancer cells while activating the ER stress response as an intrinsic protective mechanism. In addition to the negative feedback regulation of ferroptosis involved in tumor drug resistance, the activated ER stress response promotes ferroptosis development in certain diseases. Xu et al. [21] discovered ER stress signaling-mediated ferroptosis in ulcerative colitis. In the intestinal epithelial cells of mice with colitis, GRP78 and the PERK-ATF4-CHOP pathway were significantly activated. GSK414, a PERK inhibitor, significantly reduced dextran sulfate sodium-induced ferroptosis. Whole cigarette smoke condensates (WCSCs) may cause ferroptosis in bronchial epithelial cells through ER stress [90]. ER structures were damaged in 1% WCSC-treated cells. Furthermore, WCSCs clearly increased the levels of ER stress proteins (PERK, IRE-1a, Bip, and CHOP). Surprisingly, ferritin protein (light chain) expression was significantly increased near the ER.

Notably, ATF4 protein seems to be critical for ER stress-related ferroptosis. ATF4 is a stress-induced transcription factor expressed in many human organs, regulating the expression of genes involved in amino acid import, GSH biosynthesis, and oxidative stress resistance [91]. ATF4 can regulate HSPA5 expression by preventing GPX4 degradation, thereby increasing the resistance of pancreatic cancer cells to gemcitabine-induced ferroptosis [92]. Additionally, ATF4-mediated SLC7A11 upregulation in human glioma cells has been related to ferroptosis resistance [93]. Furthermore, ATF4-mediated transcriptional expression of the GSH-degrading enzyme ChaC glutathione-specific gamma-glutamylcyclotransferase 1 increases artesunate- (ART-) or cystine-induced ferroptosis in breast cancer cells [94]. ATF4 has multiple biological functions in ferroptosis because of the diversity of ATF4 target genes.

ER stress-induced Ca^2+^ release could also contribute to ferroptosis. When ROS levels increase during ferroptotic cell death, Ca^2+^ is likely released from the ER through RyR and IP3R channels [95]. Moreover, the ER-resident SOCE component Orai 1 can promote Ca^2+^ entry into cells, and blocking this component prevents glutamate-induced cell death [96].

#### 3.2.3. The Role of ER in Apoptosis–Ferroptosis Crosstalk

Changes in the ER have a role in cell fate determination. According to Hong et al. [97], ER stress may link apoptosis to ferroptosis. When combined, the apoptotic agent TRAIL synergistically interacted with ferroptotic agents such as erastin and ART to treat human pancreatic cancer PANC-1 and BxPC-3 cells and human colorectal cancer HCT116 cells. Furthermore, erastin and ART increased PUMA expression through CHOP and induced ER stress. PUMA-deficient HCT116 cells and CHOP-deficient mouse embryonic fibroblasts did not synergize with erastin/ART and TRAIL but p53-deficient HCT116 cells did. Hence, the CHOP/PUMA axis is vital for the ferroptotic agent response and may play a crucial role in ferroptotic agent-mediated sensitization to TRAIL-induced apoptosis. This suggests that ER stress acts as a link between ferroptosis and apoptosis, but the precise mechanism requires further investigation.

### 3.3. Lysosomes

#### 3.3.1. Lysosomes and Apoptosis

The lysosome, a single membrane-bound organelle, stores acid hydrolases that are involved in cellular cargo degradation and homeostasis maintenance. Cells treated with epigallocatechin gallate conjugated to bovine serum albumin undergo lysosomal expansion during apoptosis, which suggests the crucial role of lysosomes in apoptosis [98].

Cathepsin family proteases are among the most meticulously researched lysosomal hydrolases. Based on the active site amino acid (cysteine (B, C, H, F, K, L, O, S, V, and W), aspartic (D and E), or serine (G) cathepsins), they are categorized into three subgroups. Most of these proteases are believed to be closely related to apoptosis. Cathepsin B, for example, promotes apoptosis in mouse granulosa cells by activating caspase-8 and -3 [99]. Cathepsin D maturation and localization in midgut cells can activate caspase-3 and promote apoptosis [100]. In myocardial ischemia/reperfusion mice, cathepsin S inhibitors prevented an abnormal increase in cleaved caspase-8 and -3 levels [101].

Cathepsins are released into the cytosol owing to lysosomal membrane leakage or loss of lysosomal membrane integrity. Consequently, lysosomal membrane permeabilization (LMP) has an indirect but active role in apoptosis [102]. The lethal effects of LMP and cathepsins are not limited to the activation of the caspase-dependent apoptosis. LMP occurs early in the death process of small cell lung cancer cells treated with microtubule stabilizing agents. In this process, lysosomal protease cathepsin B plays a central role in inducing caspase-independent apoptosis [103]. In addition, depletion of heat shock protein 70 and moderate activation of T lymphocytes trigger LMP and cathepsin-mediated apoptosis in human-derived tumor cells, yet none of these processes occur with caspase activation [104, 105].

Various factors, including apoptosis-inducing stimuli and molecules, can cause LMP. Considerable attention has been paid to lysosomal membrane peroxidation that induces LMP [106]. Intralysosomal iron, derived from degraded metalloproteins, has been hypothesized to react with H_2_O_2_ and produce excess ROS, leading to membrane destabilization via mass peroxidation of membrane lipids. Phagocytosis of iron complexes or iron-containing proteins increases lysosomal vulnerability, whereas lowering the levels of intralysosomal reactive iron reduces lysosomal leakage and cell death [107–109].

#### 3.3.2. Lysosome and Ferroptosis

New research has indicated that ferroptosis is a type of lysosomal cell death. Pharmacological inhibition of lysosome-dependent cell death through cathepsin activity or vacuolar type H+-ATPase limits erastin-induced ferroptosis [110]. Furthermore, quercetin activates lysosomes and increases ferritin degradation, initiating ferroptosis, which indicates a role for lysosome-mediated ferritinophagy in ferroptosis [35].

Ferritinophagy is a type of autophagy wherein ferritin is used to release intracellular free iron. Nuclear receptor coactivator 4 (NCOA4) is required for transport of ferritin to lysosomes, and NCOA4-deficient cells cannot degrade ferritin, which leads to lower bioavailable intracellular iron [111]. Ferroptosis induction results in autophagy activation and subsequent degradation of ferritin and the ferritinophagy cargo receptor NCOA4. When ferritinophagy is inhibited through autophagy blockage or NCOA4 knockdown, ferroptosis-associated cellular labile iron and ROS are not accumulated, followed by the prevention of eventual ferroptosis [112]. Lipid ROS can colocalize with lysosomes. Lysosomal activity inhibitors can reduce lipid ROS burst and prevent intracellular iron provision, thereby inhibiting ferroptosis. This implies that lysosomal activity has a role in lipid ROS-mediated ferroptosis through regulation of cellular iron equilibria and ROS production [113].

Recent studies have shown that the released lysosomal cathepsins, particularly cathepsin B, are believed to mediate ferroptotic cell death. Cathepsin B translocation from the lysosome to the nucleus induces nuclear cathepsin B accumulation, which causes DNA damage and STING1-dependent ferroptosis [114]. Cathepsin B inhibition significantly improves cellular membrane integrity and chromatin degradation by preventing lysosomal membrane damage, independent of GPX4 [115].

#### 3.3.3. The Role of Lysosome in Apoptosis–Ferroptosis Crosstalk

Lysosomes are the cellular recycling center and contain numerous hydrolases with the ability to degrade most cellular macromolecules. LMP and the subsequent leakage of lysosomal content into the cytosol are key events driving lysosome-mediated cell death. LMP is primarily carried out by lysosomal cathepsin proteases and can lead to necrotic cell death, apoptosis, or ferroptosis depending on the extent of the leakage and the cellular context. Cathepsin B has a role in both apoptosis and ferroptosis. Different cathepsins may be involved in different types of cell death. Consequently, the selective release of cathepsins from lysosomes may be related to the mechanism of selection between apoptosis and ferroptosis [116]. Furthermore, lysosomal iron accumulation may be involved in the interaction between apoptosis and ferroptosis, with increased levels of lysosomal active iron during both processes [35, 108]. Lysosomal iron supplementation increases susceptibility to apoptosis and ferroptosis [107, 117], because activated iron causes lipid peroxidation in the lysosomal membrane and induces LMP.

### 3.4. GA

GA is a small membranous organelle involved in protein transport and secretion. Moreover, it acts as a stress sensor, lipid/protein modifier, mitotic checkpoint, and malignant transformation mediator in cells. The Golgi complex forms a continuous ribbon in most mammalian cells. In neurodegenerative diseases, however, the Golgi ribbon of a specific neuron group is usually broken down into isolated parts, which possibly contribute to cell death [118]. The role of GA as an apoptosis trigger has received little attention. According to current research, GA fragmentation is a crucial process in apoptosis development. Many proteins involved in Golgi structure and membrane trafficking, including golgin-160, GRASP65, giantin, GM130, and the vesicle transport protein p115, act as substrates of caspases [119]. Among them, golgin-160 is a receptor for caspase-2, which is found on the Golgi membrane and can hydrolyze golgin-160. At an early apoptosis stage, caspase-3 specifically cleaves GRASP65, a Golgi reassembly and stacking protein. The Golgi vesicle transport protein p115 is cleaved early in apoptosis, prior to visible Golgi fragmentation. Its 205 amino acid C-terminal fragment influences transcription and promotes apoptosis [120].

GA stress is an autoregulatory mechanism activated in response to increased demand in Golgi functions. It may be related to both apoptosis and ferroptosis. When GA abundance and capacity are insufficient compared to cellular demand, the GA stress response is activated to improve GA function. In mammals, this response involves the activation of the TFE3, heat shock protein 47 (HSP 47), CREB3, E26 transformation specific (ETS), proteoglycan, and mucin pathways. The CREB3 and ETS pathways, for example, can promote apoptosis [121]. Golgi stress inducers caused ferroptosis in HeLa cells, which can be prevented through SLC7A11 or GPX4 overexpression, as well as ACSL4 depletion [122]. Additionally, ferroptosis inhibitors can prevent cell death, protect against Golgi dispersal, and inhibit protein secretion in response to several GA stress agents. The precise mechanism of GA stress in the apoptosis–ferroptosis crosstalk, however, remains unknown.

### 3.5. Peroxisomes

As the needs of the cell and the varying environments, peroxisomes carry out numerous tightly controlled oxidative reactions. Interestingly, they seem to have a dual role in apoptosis and ferroptosis. Peroxisomes, as a critical ROS rheostat, protect lymphoma cells from vorinostat-mediated apoptosis [123]. On the other hand, they trigger ferroptosis by synthesizing PUFA ether phospholipids, which act as substrates for lipid peroxidation [124]. This could be an organelle antagonistic mechanism when determining the cell fate.

### 3.6. Organelle Interactions

A regulatory crosstalk between different subcellular organelles is required for cell homeostasis maintenance as well as for inducing cell death. Interactions between the ER and mitochondria, which occur in both apoptosis and ferroptosis, are the most well-studied organelle interactions. ER stress is involved in apoptosis, and the Bcl-2 family, as mitochondrial-level central regulators of apoptosis, is almost always involved in the regulation [125]. WCSC causes ferroptosis in bronchial epithelial cells through ER stress and disrupts mitochondrial homeostasis [90]. These studies have suggested that ER stress can cause cell death by affecting mitochondria, possibly because the ER and mitochondria communicate via mitochondria-associated ER membranes (MAMs). MAMs regulate lipid synthesis, Ca^2+^ signaling, and intracellular trafficking. Importantly, MAMs also participate in the regulation of apoptosis and ferroptosis. Ca^2+^, the ubiquitous second messenger that regulates numerous physiological events, is a key player. ER-to-mitochondrion Ca^2+^ transfer via MAMs is involved in apoptosis regulation [126]. Similarly, mitochondrial Ca^2+^ uptake regulator 1 (MICU1) was found to regulate cold stress-induced ferroptosis in A549 cells [127]. Despite the paucity of research on MAMs and ferroptosis, Ca^2+^ release from the ER into the cytoplasm increases during ferroptosis [95]. If uncontrolled, the increasing cytoplasmic Ca^2+^ levels may cause mitochondrial Ca^2+^ overload, possibly contributing to ferroptosis-induced neuronal damage [128].

Apoptosis and ferroptosis are also supported by the cooperation of the lysosomes and mitochondria. Myocardial infarction causes abnormal mitochondria–lysosome contacts, leading to lysosomal enlargement, impaired lysosomal clearance of damaged mitochondria, and cardiomyocyte apoptosis [129]. Lysosomal enzymes can activate proapoptotic BID and degrade antiapoptotic proteins, thus triggering a mitochondria-dependent cell death pathway [130]. Moreover, when cathepsin B is inhibited, AIF translocation from the mitochondria to the cytosol/nucleus and then ferroptosis are inhibited, implying that LMP precedes and mediates mitochondrial structure damage, promoting ferroptosis through cathepsin B leakage or excessive ROS production following lysosomal damage [115]. Notably, carbonic anhydrase 9 inhibition causes a mixed type of cell death including ferroptosis and apoptosis along with mitochondrial fission and enhanced autophagy with increased catalytic Fe^2+^ in both the mitochondria and lysosomes. This implies a potential role for cooperative interaction between the mitochondria and lysosomes in the ferroptosis–apoptosis crosstalk [131].

Some organelle interactions have been reported in apoptosis but not in ferroptosis. For example, GD3 synthase, present in the Golgi membranes, participates in the conversion of ceramide into GD3, which induces mitochondrial permeability transition, the mitochondrial apoptosis pathway, and CD95 transport from the GA to the plasma membrane [132]. Under basal conditions, peroxisome deficiency increases cytosolic Cyt c and caspase activities without inducing apoptosis [133]. It also significantly increases etoposide-induced caspase activation and apoptosis, thereby indicating augmented cellular sensitivity to death signals. Moreover, GalNAc-bn treatment triggers Golgi disassembly; nuclei splitting; activation of the Golgi-resident caspase-2, ER stress-related molecules, and mitochondria-resident caspase-9; and the efflux of Cyt c from the mitochondria to the cytosol through MOMP, eventually leading to apoptosis of HSP47 knockdown cells; this indicates that Golgi stress can influence the ER and mitochondria [134].

## 4. Future Prospects

Various cell death-inducing conceptional therapies have been extensively researched for cancer treatment. Most tumor cells are expected to die due to apoptosis. However, apoptosis evasion and enhanced antiapoptosis ability are currently regarded as the primary causes of tumor refractoriness. In this case, ferroptosis has received extensive academic attention as a PCD type that is different from apoptosis.

Various therapeutic agents are currently being discovered and synthesized to synergize apoptosis and ferroptosis against cancer [135–137]. The cytotoxic effects of cisplatin, a powerful anticancer agent widely used against solid tumors, were believed to induce apoptosis by generating nuclear DNA adducts. Nonetheless, a recent study [138] revealed that cisplatin induces both ferroptosis and apoptosis in A549 and HCT116 cells and that the combination of cisplatin and the ferroptosis inducer erastin had a significant additive effect on antitumor activities. Variations in drug cellular accumulation, DNA repair, and cytosolic inactivation all affect cisplatin-induced apoptosis and trigger resistance to cisplatin but not ferroptosis, which implies that ferroptosis can complement apoptosis in antitumor therapy. Furthermore, a metal–organic network can switch apoptosis to ferroptosis, thereby mediating high-efficiency anticancer therapy [139].

Some studies have found that organelles play a crucial and multifunctional role in tumor progression [140, 141]. In addition, many studies mentioned in this paper show that different organelles and their interactions play an important function in the apoptosis–ferroptosis crosstalk. Therefore, huge therapeutic opportunities open up by targeting organelles. Ferroptosis may act as a novel approach for cancer treatment, and converting apoptosis to ferroptosis by targeting cellular organelles may help address tumor resistance in a novel way.

## 5. Conclusions

**Figure 2:**
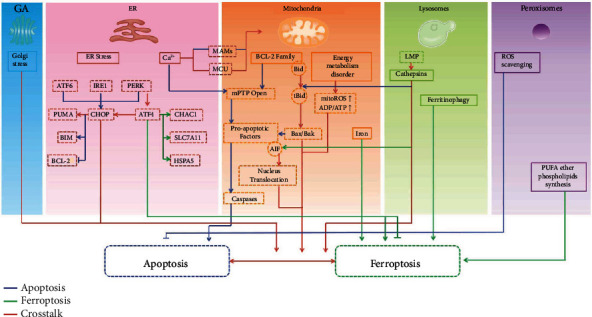
Known organelle-specific mechanisms involved in apoptosis and ferroptosis.

Emerging evidence suggests that different cell death types have many common molecular effectors and signaling pathways rather than distinct boundaries. Two such cell death modes, apoptosis and ferroptosis, both involve multiple organelle dysfunctions, which can be viewed as a link between the two (Figure 2). However, their roles in regulating the crosstalk between these two processes remain unknown. When different specifically altered signals are detected, a series of biochemical events are triggered that lead to different cell fates, which is possibly related to the presence of antagonistic or selective mechanisms between apoptosis and ferroptosis. Because the role of organelles in ferroptosis is poorly understood, additional studies are warranted to allow for a more in-depth investigation of this PCD type at the subcellular level.

## Figures and Tables

**Table 1:** Distinct modalities of apoptosis and ferroptosis.

	Apoptosis	Ferroptosis
Morphological characteristics	Cell membrane: blebbing of the plasma membrane and cell rounding Cytoplasm: pseudopod retraction and cellular volume reduction Nucleus: Nuclear fragmentation, nuclear volume reduction, and chromatin condensation	Cell membrane: absence of plasma membrane rupture and blebbing and cell rounding Cytoplasm: small mitochondria with condensed mitochondrial membrane densities, mitochondrial crista reduction or disappearance, and outer mitochondrial membrane rupture Nucleus: normal nuclear size and lack of chromatin condensation
Biochemical features	Activation of caspases and oligonucleosomal DNA fragmentation	Inhibition of system Xc- and GPX4, reduced GSH, and iron accumulation and lipid peroxidation
Key genes	Caspase, p53, Fas, Bcl-2, and Bax	SLC7A11, GPX4, Nrf2, and TFR1
Inducers and inhibitors	Inducers: FASL and UNC5B Inhibitors: XIAP, NAIP, and Z-VADFMK	Inducers: erastin, RSL3, sorafenib, and acrolein Inhibitors: DFO, Fer-1, liproxstatin-1, vitamin E, and carvacrol

GPX4: glutathione peroxidase-4; GSH: glutathione; Bcl-2: B-cell lymphoma-2; SLC7A11: solute carrier family 7 member 11; Nrf2: NF-E2-related factor 2; FASL: Fas ligand; UNC5B: uncoordinated 5B; XIAP: X-linked inhibitor of apoptosis; NAIP: NOD-like receptor family apoptosis inhibitory protein; DFO: deferoxamine; Fer-1: ferrostatin-1.

**Table 2:** Known morphological and functional changes in subcellular organelles during apoptosis and ferroptosis.

Organelle	Apoptosis	Ferroptosis	Commonalities
Mitochondria	Proapoptotic factor release ↑	Swollen mitochondria Decrease in cristae Mitochondrial iron ↑	Mitochondrial membrane potential ↓ Mitochondrial membrane permeability ↑ Mitochondrial energy metabolism disorder Mitochondrial ROS ↑ Mitochondrial Ca^2+^ ↑
ER	MAMs ↑	Viscosity of ER ↑	ER stress ↑ Release of Ca^2+^ from the ER ↑
Lysosomes	Lysosomal expansion	Ferritinophagy	Lysosomal cathepsins ↑ LMP ↑ Lysosomal iron ↑
GA	GA fragmentation	Golgi dispersal	Golgi stress ↑
Peroxisomes	ROS scavenging ↑	PUFA ether phospholipid synthesis ↑	Unknown

ER: endoplasmic reticulum; ROS: reactive oxygen species; MAMs: mitochondria-associated endoplasmic reticulum membranes; LMP: lysosomal membrane permeabilization; GA: Golgi apparatus; PUFA: polyunsaturated fatty acids.

## Data Availability

The data that support the findings of this study are available from the corresponding author, Lei Zhao, upon reasonable request.
